# Tempo and mode of evolutionary radiation in Diabroticina beetles (genera *Acalymma*, *Cerotoma*, and *Diabrotica*)

**DOI:** 10.3897/zookeys.332.5220

**Published:** 2013-09-19

**Authors:** Astrid Eben, Alejandro Espinosa de los Monteros

**Affiliations:** 1 Julius Kühn-Institut, Federal Research Centre for Cultivated Plants JKI, Schwabenheimer Straße 101, 69221 Dossenheim, Germany; 2 Departamento de Biología Evolutiva, Instituto de Ecología, AC. Carretera Antigua a Coatepec # 351, 91070 Xalapa, Veracruz, México

**Keywords:** Coalescence time, Diabroticina, host plants range, macroevolution, pharmacophagy, phylogeny

## Abstract

Adaptive radiation is an aspect of evolutionary biology encompassing microevolution and macroevolution, for explaining the principles of lineage divergence. There are intrinsic as well as extrinsic factors that can be postulated to explain that adaptive radiation has taken place in specific lineages. The Diabroticina beetles are a prominent example of differential diversity that could be examined in detail to explain the diverse paradigms of adaptive radiation. Macroevolutionary analyses must present the differential diversity patterns in a chronological framework. The current study reviews the processes that shaped the differential diversity of some Diabroticina lineages (i.e. genera *Acalymma*, *Cerotoma*, and *Diabrotica*). These diversity patterns and the putative processes that produced them are discussed within a statistically reliable estimate of time. This was achieved by performing phylogenetic and coalescent analyses for 44 species of chrysomelid beetles. The data set encompassed a total of 2,718 nucleotide positions from three mitochondrial and two nuclear loci. Pharmacophagy, host plant coevolution, competitive exclusion, and geomorphological complexity are discussed as putative factors that might have influenced the observed diversity patterns. The coalescent analysis concluded that the main radiation within Diabroticina beetles occurred between middle Oligocene and middle Miocene. Therefore, the radiation observed in these beetles is not recent (i.e. post-Panamanian uplift, 4 Mya). Only a few speciation events in the genus *Diabrotica* might be the result of the Pleistocene climatic oscillations.

## Introduction

Why does a clade have more species than others within the same lineage? This is a common question in evolutionary biology that has been pondered for almost a century (Simson 1953, Givnish and Sytsma 1997, Schluter 2000, Harmon et al. 2003). The phylogenetic pattern of cladogenesis that often is accompanied by ecological and/or morphological disparity among lineages is known as “evolutionary radiation”. The study of the processes and patterns in evolutionary biology are structured in three hierarchical levels of complexity: population level (microevolution), species level (speciation and biodiversity), and supraspecific level (macroevolution). The latter level usually explores patterns of diversity between and within monophyletic lineages. In phylogenetic analysis, macroevolution is also viewed as the origin of mayor phenotypic characters or character complexes (i.e. key characters) that permit a lineage to undergo an adaptive radiation (Nitecki 1990). In this sense, macroevolution assesses the level of morphological divergence and their presumed adaptive outcome. Macroevolution can be focused on taxon patterns such as lineage richness (differential species diversity), and/or character patterns (Cracraft 1982). Macroevolutionary analyses should present such patterns in a chronological framework; otherwise, they are meaningless for explaining biodiversity scenarios. The optimal way to do so is to explain the pattern in the light of a cladistic hypothesis for the group in question. Consequently, macroevolutionary conclusions must be linkedto a phylogenetic hypothesis for three main reasons. First, only monophyletic lineages have evolutionary meaning. Second, cladogenesis is inferred; therefore, biodiversity can be quantified. Third, the origin of key characters can be established; thus, they can be correlated to biodiversity differences.

Nevertheless, there are examples in which lineage radiation is not necessarily correlated with the evolution of phenotypic characters (Farrell and Mitter 1994, Janson et al. 2008, Tilmon 2008, Ramamurthy and Gaur 2012). Observed diversity patterns, such as radiation or large-scale macroevolutionary trends, are the balance between speciation and extinction rates that can be modulated also by extrinsic factors (e.g. environmental complexity). It is also important to consider that speciation is not necessarily an adaptive process (Cracraft 1985). The changes of biodiversity at different dimensions can be the outcome of differential sorting of species. Environmental factors may determine the range of resource use between populations; therefore, affecting reproduction and mortality rates. In fact, extrinsic physical factors, such as geological or climatic history, possibly have a stronger effect in modulating global speciation and extinction rates than the emergence of key features in taxa.

The family Chrysomelidae is the most species rich lineage of Coleoptera with nearly 40,000 described species. All species feed on plants, and most of the species are specialists on a certain host (Jolivet and Hawkeswood 1997). Diabroticina beetles are a neotropical lineage. One species, *Diabrotica virgifera virgifera* LeConte, however, has recently been introduced to Europe where it has quickly become a pest on corn (*Diabrotica virgifera virgifera*) (www.eppo.int). Females lay their eggs in the ground and larvae feed on roots of the host plant, whereas adults feed on leaves and pollen. The subtribe Diabroticina encompasses 933 species (sensu Seeno and Wilcox 1982) distributed primarily in Mesoamerica and Brazil (Branson and Krysan 1981). It is divided in the sections Diabroticites, Cerotomites, Phyllecthrites and Trachyscelites. Webster (1895) concluded that the center of origin of these beetles was Mesoamerica based on the diversity and species richness. However, the information on the natural history of Diabroticina is scarce, and for many species nonexistent. Most of what we know about these insects has been derived from less than 70 species, most of them belonging to the genera *Acalymma* and *Diabrotica*. All the 72 recognized species of *Acalymma* are specialists on Cucurbitaceae. The *Diabrotica*, the most diverse genus within the subtribe, has been traditionally divided in three groups (i.e. polyphagous fucata with more than 300 species, oligophagous virgifera that encompass 24 species, and signifera with only 11 species). Signifera is endemic to South America, no pest species have been reported, and their biology is almost unknown.

The aim of the present study is to review the processes that shaped the speciation pattern of some lineages encompassed in Diabroticina beetles (i.e. genera *Acalymma*, *Cerotoma*, and *Diabrotica*), and to set those processes within a reliable time framework. To reach this objective we have performed phylogenetic and coalescent analyses based on DNA sequences from mitochondrial and nuclear loci. In a previous study we applied a molecular clock hypothesis on the evolutionary scenario for host-range expansion in Diabroticinas (Eben and Espinosa de los Monteros 2008). For that scenario, however, only a few nodes were dated. Moreover, we used a strict-clock model on trees that became ultrametric after pruning the lineages that did not pass the constant-rate test. The novelty of the present study is that the phylogeny is entirely dated. These dates are inferred based on more reliable coalescent models that can independently handle unlinked molecular partitions, and account for rate-variations among lineages. Furthermore, here we discuss, in addition to host-range interactions, other processes that may affect the speciation rate of these insects.

## Materials and methods

### Diabroticina beetles and molecular markers

DNA sequences for 44 lineages of chrysomelid beetles were used for this study (Table 1). Most taxa were chosen because they occur sympatrically in Mexico that has been proposed as the putative centre of origin for Diabroticites. Twenty-four species were collected in the field and sequenced by us. The remaining taxa encompassed in the dataset were selected based on DNA sequences availability.

**Table 1.** Diabroticina specimens used and GenBank accession numbers for the molecular markers.<br/>

**Taxa**	**COI**	**12S rRNA**	**16S rRNA**	**28S rRNA**	**ITS2**
*Acalymma albidovittatum* Baly	AY242447*	AY243713*			
*Acalymma bivittatum* (Fabricius)	AY242443*	AY243709*			
*Acalymma blandulum* LeConte	AF278543^	AF278558^			
*Acalymma blomorum* Munroe & Smith	**AY533582**	**AY533610**	**AY533637**	AY243710*	
*Acalymma fairmairei* (Fabricius)	**AY533583**	**AY533611**	**AY533638**	AY243708*	
*Acalymma innubum* (Fabricius)	**AY533585**	**AY533613**	**AY533640**		
*Acalymma trivittatum* Mannerheim	**AY533584**	**AY533612**	**AY533639**	AY243711*	
*Acalymma vittatum* (F.)	**AY533586**	**AY533614**	**AY533641**	AY646317*	AF278557^
*Amphelasma cavum* (Say)	**AY533590**	**AY533618**	**AY533645**		
*Amphelasma nigrolineatum* Jacoby	AY242488*	AY243754*			
*Amphelasma sexlineatum* Jacoby	AY242489*	AY243755*			
*Cerotoma arcuata* Olivier	AY242494*	AY243760*			
*Cerotoma atrofasciata* Jacoby	**AY533587**	**AY533615**	**AY533642**		
*Cerotoma fascialis* Erickson	AY646323*				
*Cerotoma ruficornis* Olivier	AY646322*				
*Cerotoma trifurcata* (Forster)	AF395803				
*Diabrotica adelpha* Harold	AF278552^	AY243735*	AF278567^		
*Diabrotica amecameca* Krysan & Smith	**AY533578**	**AY533606**	**AY533634**		
*Diabrotica balteata* LeConte	**AY533569**	**AY533597**	**AY533625**	AY243731*	AF278568^
*Diabrotica barberi* Smith & Lawrence	AF278544^	AF278559			
*Diabrotica biannularis* Harold	AY242466°	AY243732*			
*Diabrotica cristata* (Harris)	**AY533580**	**AY533608**	AF278560^		
*Diabrotica decempunctata* Latreille	AY242467°	AY243733*			
*Diabrotica dissimilis* Jacoby	**AY533577**	**AY533605**	**AY533633**		
*Diabrotica lemniscata* LeConte	AF278546^	AF278561^			
*Diabrotica limitata* (Sahlberg)	AY242481°	AY243747*			
*Diabrotica longicornis* (Say)	AF278547^	AF278562^			
*Diabrotica nummularis* Harold	**AY533568**	**AY533596**	**AY533624**		
*Diabrotica porracea* Harold	**AY533571**	**AY533599**	**AY533627**	AY243737*	AF278563^
*Diabrotica scutellata* Baly	**AY533567**	**AY533595**	**AY533623**		
*Diabrotica sexmaculata* Baly	**AY533566**	**AY533594**	**AY533622**		
*Diabrotica speciosa* Germar	**AY533579**	**AY533607**	**AY533635**	AY646319*	AF278569^
*Diabrotica tibialis* Baly	**AY533576**	**AY533604**	**AY533632**	AY243746*	
*Diabrotica undecimpunctata duodecimnotata* Harold	**AY533572**	**AY533600**	**AY533628**		
*Diabrotica undecimpunctata howardi* Barber	**AY533573**	**AY533601**	**AY533629**	AY243738*	AF278570^
*Diabrotica undecimpunctata undecimpunctata* Barber	AF278556^	AF278571^			
*Diabrotica virgifera virgifera* LeConte	**AY533575**	**AY533603**	**AY533631**	AY243734*	AF278564
*Diabrotica virgifera zeae* Krysan & Smith	**AY533574**	**AY533602**	**AY533630**	AF278565	
*Diabrotica viridula* (Fabricius)	**AY533570**	**AY533598**	**AY533626**	AY243748*	AF278566
*Paratriarius curtisii* Baly	**AY533591**	**AY533619**			
*Paratriarius subimpressa* Jacoby	AY242461°	AY243727*			
*Trichobrotica nymphaea* (Jacoby)	AY242440°	AY243706*			
*Trichobrotica sexplagiata* Jacoby	**AY533581**	**AY533509**	**AY533636**		
*Schematiza flavofasciata* Guér	AY515035^+^	AY507265^+^	**EF197976**	AY243786*	AY514312^+^

^: Clark et al. 2001;

**bold**: Eben and Espinosa de los Monteros 2008;

°: Gillespie et al. 2003;

*: Gillespie et al. 2004;

+: Swigonova and Kjer 2004.

Three mitochondrial-genome regions (i.e. COI, 12S, and 16S) were sequenced to provide the adequate level of variability for reconstructing the phylogeny of this group. To complement the molecular dataset we downloaded supplementary data available in GenBank. This database provided us with two additional nuclear fragments (i.e. 28S and ITS2) and 20 extra Diabroticina species. The concatenated matrix, therefore, included sequences from five loci (three mitochondrial, and two nuclear), encompassing 44 taxa and 2,718 nucleotide positions. Sequences for the 28S and complementary sequences for the cytochrome oxidase subunit I (COI) gene were taken from studies by Gillespie et al. (2003, 2004). Sequences of the internal transcribed spacer 2 (ITS2) were taken from Clark et al. (2001). The use of alternative sources of molecular data has the inconvenience that taxon sampling differs among authors. As a consequence the full matrix contains missing entries (Table 1). The core ingroup encompassed 34 Diabroticite lineages. As putative outgroups we included five species from the genus *Cerotoma* (Cerotomites), and two species from the genus *Trichobrotica* (Phyllecthrites). To root the phylogenetic hypothesis we employed the sequences published by Swigonova and Kjer (2004) for *Schematiza flavofasciata*. We selected the outgroup species based on the phylogeny presented by Gillespie et al. (2003). The entire species list and the GenBank accession numbers for the molecular markers are provided in Table 1.

### DNA extraction, PCR, and sequencing techniques

A small amount of tissue (i.e. 2 or 3 legs) was ground in Chelex 5% (w/v solution) for total genomic DNA extraction following the method suggested by Singer-Sam et al. (1989). Oligonucleotides specifically designed for beetles were used for DNA amplification. PCR assays were conducted in Peltier-effect thermocyclers (ABI GeneAmp PCR system 2400) using the following parameters: one initial cycle at 95° C for 120 s, followed by 30 cycles of 95° C for 20 s, 50° C for 20 s, 72° C for 60 s, with one final cycle at 72° C for 180 s. All PCR reactions were conducted along with positive and negative controls to detect potential false positives due to contamination. Successful amplifications were purified using the UltraClean TM 15 DNA Purification kit (MoBio Laboratories Inc.). Purified PCR products were subjected to cycle sequencing using the ABI Prism BigDye® Terminator v 3.1 Cycle Sequencing Kit, following the protocol suggested in the kit instructions. The excess of *Taq* dideoxy terminators was removed with Centri-Sep spin columns (Princeton Separations) in a variable speed microcentrifuge at 2 500 rpm for 2 min. Final purifications were dried down in a vacuum centrifuge and suspended in 25 µl of the loading solution. Sequencing products were subjected to capillary electrophoresis in the ABI Prism 310 DNA Sequencer (Perkin Elmer). Sequence files were analyzed with the aid of the program SEQUENCHER v 4.0 (Gene Codes Corp., Ann Arbor, MI). Fragments were sequenced on both DNA strands to ensure accurate data collection.

### Phylogenetic reconstruction and temporal scenario

The phylogenetic hypothesis was reconstructed using Bayesian inference (BI). We used an Akaike Information Criterion (Alfaro and Huelsenbeck 2006) in jMODELTEST v 2.0.2 (Posada 2008) to select an appropriate model of nucleotide substitution for each locus and the concatenated dataset. Table 2 presents the best-fit models selected and the specific parameters that were incorporated as prior information in the BI analyses. These were performed on each molecular marker as well as on the combined dataset using MRBAYES v. 3.2.1 (Ronquist and Huelsenbeck 2003). Two sets of analyses were performed for the combined dataset. The first used a single model for the entire combined loci dataset (the “unpartitioned” analyses) and the second set of analyses employed partition-specific DNA evolution models of each gene. For each dataset, two parallel Markov chain Monte Carlo (MCMC) analyses were executed simultaneously, and each was run for a minimum of 20 million generations, sampling every 1 000 generations. A majority consensus tree was calculated, showing nodes with a posterior probability (*PP*) of 0.5 or more. Bayesian posterior probability values were calculated from the sampled trees remaining after 25% burn-in samples were discarded (Ronquist and Huelsenbeck 2003) to only include trees after the –lnL scores reached an asymptote. The consensus tree was drawn with FIGTREE v 1.3.1 (http://tree.bio.ed.ac.uk/software/figtree/). We used Bayes factors to determine whether applying partition-specific models significantly improved explanation of the data (Nylander et al. 2004).

**Table 2.** Molecular markers best-fit evolutionary model, model parameters, and mean likelihood for trees inferred from Bayesian analyses.<br/>

**Maker**	**Model**	**Nucleotide frequency**	Γ	**Rate matrix**	**p-inv**	**lnL HM**
12S rRNA	TPM1uf + Γ	0.380, 0.050, 0.117, 0.453	0.477	1.00, 5.29, 1.76, 1.76, 5.29, 1.00	nr	-2158.86
16S rRNA	TVM + Γ	0.409, 0.161, 0.080, 0.350	0.245	0.94, 4.25, 3.70, 0.83, 4.25, 1.00	nr	-2622.66
28S rRNA	TPM2 + I	0.250, 0.250, 0.250, 0.250	nr	2.50, 9.48, 2.50, 1.00, 9.48, 1.00	0.759	-2001.79
COI	GTR + Γ + I	0.341, 0.133, 0.104, 0.422	0.384	0.80, 6.38, 2.05, 0.92, 17.7, 1.00	0.416	-7899.90
ITS2	TPM1uf + Γ	0.299, 0.180, 0.210, 0.312	0.411	1.00, 3.86, 1.64, 1.64, 3.86, 1.00	nr	-1891.23
Total evidence	GTR + Γ + I	0.313, 0.157, 0.171, 0.359	0.625	1.32, 6.07, 4.10, 1.02, 10.2, 1.00	0.535	-17525.05

lnL HM = harmonic mean of the normal logarithm for the tree likelihood score;

nr = not relevant in the best-fit model.

Times for potential isolation events within the Diabroticina beetles' phylogeny were calculated using the software BEAST v 1.7.5. (Drummond and Rambaut 2007). The trees inferred from the phylogenetic analyses were used to constrain specific monophyletic groups. For dating we used the five data partitions (i.e. 12S, 16S, 28S, COI, and ITS2), each with independent evolutionary models as chosen by jMODELTEST. Substitution rates of the five genes were unlinked to be estimated independently. However, calibrating the tree is difficult and involves making a number of assumptions (Burleigh 2012). Based on both, fossil records and secondary calibrations inferred from isozyme studies, Metcalf (1986) suggested that the splitting event between the genus *Diabrotica* and the genus *Acalymma* occurred approximately 45 million years ago (Mya). As far as we know, this is the most reliable published date for the divergence time within the Diabroticina lineage. We, therefore, used Metcalf's data to estimate nucleotide evolution rates for each molecular marker. Based on the mean genetic distance between the genera *Diabrotica* and *Acalymma* we decided to set the follow nucleotide substitution rates: 0.00253 my^–1^ for the 12S, 0.0024 my^–1^ for 16S, 0.0006 my^–1^ for 28S, 0.004 my^–1^ for COI, and 0.002 my^–1^ for ITS2. The “relaxed clock (uncorrelated)” model was used with a lognormal distribution of rates. The Markov chain Monte Carlo was run 10 times for 10 million generations, and parameters were sampled every 1000th generations. The program TRACER v 1.5 (Rambaut and Drummond 2007) was used for assessing stationarity of the McMC, effective sample sizes (ESSs), and posterior intervals spanning the 95% highest posterior density. The single runs were combined with LogCombiner implemented in the BEAST package. Trees were summarized using TREEANOTATOR v 1.6.1, and displayed in FIGTREE v 1.3.1.

Finally, we compared the rate and timing of diversification events among the major lineages of Diabroticina. The average time between nodes was used as a straightforward measurement of speciation time. It was obtained directly from the chronogram inferred with BEAST. We also calculated the D and S indexes that describe diversification rate (Good-Avila et al. 2006). The former is based on a pure-birth model for the rate of diversification, whereas the latter assumes a constant rate of speciation but uses the phylogenetic information in the tree (i.e. branch length). Both indexes give the diversification rate in species per million years, and allow for simple comparisons among groups. To assess changes in diversification rates within genera we computed the γ statistic (Pybus and Harvey 2000). Under a constant speciation rate, γ has a standard normal distribution. However, it becomes negative when speciation has occurred more frequently early in the lineage history (deceleration of diversification rate), or positive when speciation has occurred more frequently toward the present (acceleration of diversification rate). This statistic was computed with the aid of the package APE v3.0-8 (Paradis et al. 2004) written in R language.

## Results

### Phylogeny

**Figure 1.**
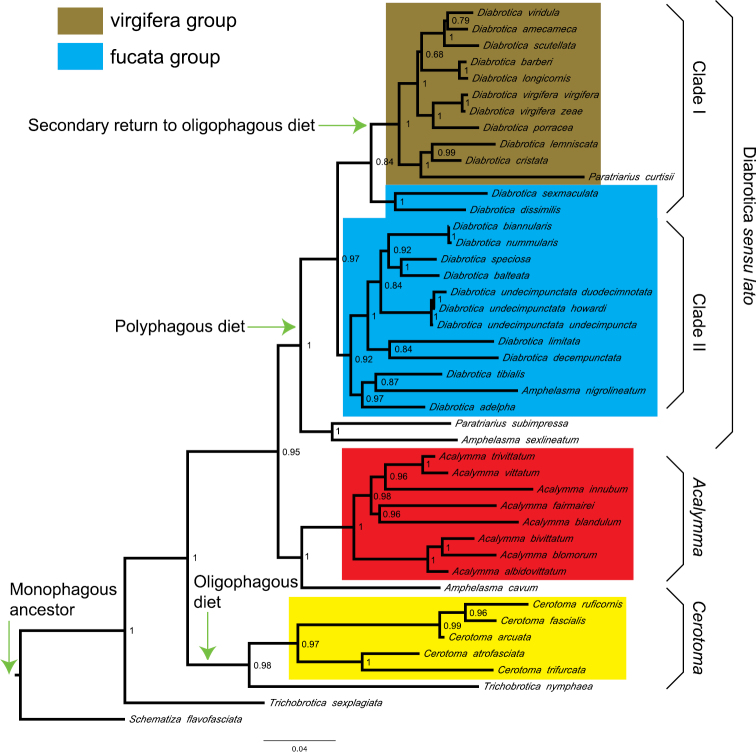
Phylogenetic tree recovered from Bayesian inference showing posterior provability values at the nodes. The genera *Acalymma* and *Cerotoma* are recovered as monophyletic lineages. *Diabrotica*, however, is paraphyletic unless some species of *Amphelasma* and *Paratriarius* are renamed as *Diabrotica*. The general evolutionary scenario for changes in diet spectrum is mapped in the phylogeny.

The topologies of the best-scoring trees obtained for the individual partitions were congruent with the concatenated tree, with most nodes having good support (Figure 1). The Bayes factor indicated that the BI tree obtained with the data partitioned by DNA region was more informative (2ln = 7.48), although this difference was not necessarily significant (Kass and Raftery 1995). Phylogenetic relationships of major groupings represented in our study are supported with *PP* values ≥ 0.95 and their phylogenetic relationships were largely consistent with previous phylogenetic studies (Clark et al. 2001, Gillespie et al. 2003, Eben and Espinosa de los Monteros 2004). Likewise, the phylogenetic analyses in MrBayes inferred from the individual markers yielded congruent inter-specific relationships with strong support (not shown, available upon request from the authors), including the same general interrelationships within *Diabrotica*. Our study suggests that the genera *Amphelasma* and *Paratriarius* are paraphyletic, with *Paratriarius curtisii* being more closely related to the virgifera group; whereas *Amphelasma nigrolineatum* is intermixed with *Diabrotica* species that belong to the fucata group (Figure 1). Two species of *Amphelasma* (i.e. *Amphelasma nigrolineatum*, *Amphelasma sexlineatum*) in addition tothe two species of *Paratriarius* (i.e. *Paratriarius curtisii*, *Paratriarius subimpresa*) were found in different clades; therefore, these genera apparently are not monophyletic. Those species, nonetheless, are more closely related to the genus *Diabrotica* than to any other genus. From now on, therefore, we will refer to *Diabrotica* *sensu lato*, including all the species encompassed in the genus *Diabrotica*, the two species of *Paratriarius* and the two species of *Amphelasma*. The third species of *Amphelasma* included in this study (*Amphelasma cavum*) was recovered far from the other members of this genus, as the sister taxon of the genus *Acalymma* forming a highly supported clade (*PP* value = 1).

In the strict sense, the proposal for subdividing the *Diabrotica* species into smaller groups (i.e. virgiferaandfucata) was not supported in our study (Figure 1). Among the strongly supported relationships for *Diabrotica* *sensu lato* are the following: a) *Diabrotica* *sensu lato* was found to be monophyletic (*PP* = 1.0) and sister to the *Amphelasma cavum*-*Acalymma* spp. clade; b) *Diabrotica* *sensu lato* is split into three distinct monophyletic clades; c) all the species belonging to the so-called virgifera group are encompassed in Clade I in a well supported apical clade (*PP* = 1.0); d) in spite of that, the virgifera group is not supported as monophyletic group, since *Paratriarius curtisii* is included within this major clade; e) in Clade I, sister to the virgifera species is a monophyletic clade form by two fucata beetles (i.e. *Diabrotica dissimilis* and *Diabrotica sexmaculata*); f) the heterogeneity and instability of Clade I is demonstrated by the low support scored by BI (*PP* = 0.84); g) with moderate support (*PP* = 0.92) Clade II encompasses most of the species usually placed within the so-called fucata group (*Diabrotica adelpha* and *Diabrotica tibialis*, nonetheless, are found to be more closely related to *Amphelasma nigrolineatum* than to the remaining fucata species); h) at the base *Diabrotica* *sensu lato*, and sister to clades I-II, we recovered a highly supported (*PP* = 1.0) monophyletic clade form by *Amphelasma sexlineatum* and *Paratriarius subimpressa*. The polyphyly of the fucata group has been acknowledged before. Fucata was created as a convenience group for hosting a large number of highly variable species that did not fit into the virgifera or signifera group. Notwithstanding, the fucata and virgifera group could be easily rescued. Small adjustments, such as renaming *Paratriarius* to *Diabrotica*, and reassigning species from fucata into virgifera, would reconcile the observed phylogenetic pattern with the traditional taxonomy.

The other two genera survey within our analysis showedphylogenetic patterns more consistent with the taxonomic schemes. All the species of *Acalymma* form a strongly supported monophyletic clade (*PP* = 1.0). The *Acalymma* clade is divided in two monophyletic groups: one presents a strong interrelationship between *Acalymma bivittatum*-*Acalymma blomorum*, and *Acalymma albidovittatum*; whereas the other showed the next cladistic structure ((*Acalymma blandulum*, *Acalymma fairmairei*), (*Acalymma trivittatum*, *Acalymma vittatum*), *Acalymma innubum*). The genus *Cerotoma* is also monophyletic (*PP* = 0.97), and forms a sister genus to the Phyllecthrites species *Trichobrotica nymphaea*. This clade, formed by *Cerotoma* spp.-*Trichobrotica nymphaea*, is the most basal monophyletic group, and is the sister group of the “Acalymma-Diabrotica” clade (Figure 1).

### Divergence time estimates

**Figure 2.**
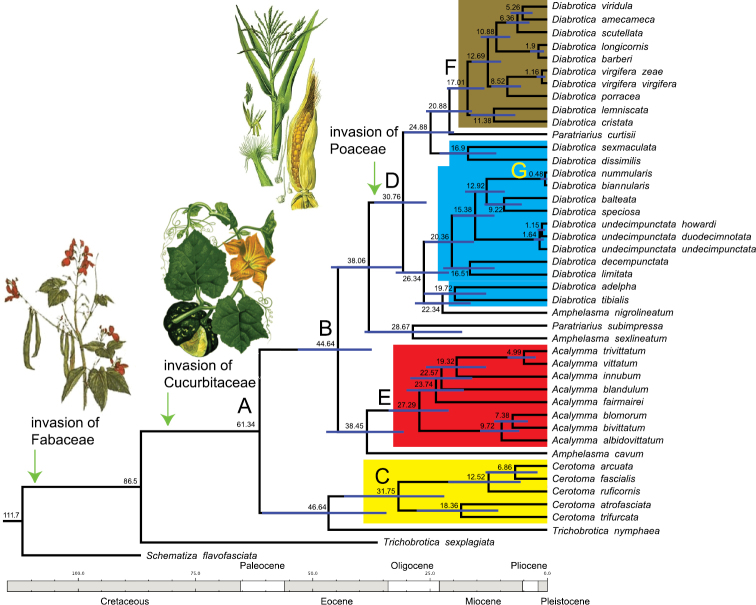
Chronogram inferred from a coalescence analysis. The blue lines at the nodes indicate the 95% confidence range for the estimated split times. Letters A to G pinpoint at key nodes in the evolutionary history of Diabroticina beetles (see Table 3 for further detail). The evolutionary scenario for the acquisition of main plant hosts is presented.

The comparison of all coalescence analyses revealed high convergence among the inferred parameters, and ESSs were larger than 200 for all of them. Analyses of divergence time estimation using the calibration method resulted in very similar divergence estimates, for both the concatenated (Figure 2, Table 3) and the individual matrices (not shown; but available upon request). Likewise, the BEAST analyses based on the concatenateddataset, using the coalescent method assuming constant population size, exponential growth or logistic growth yielded similar time estimates for the different nodes of major Diabroticina clades. These results can thus be considered as robust (Figure 2, Table 3).

**Table 3.** Chronology for key events during the evolutionary history of Diabroticina beetles.<br/>

**Node** *	**Event**	**Time Inferred**	**95% confidence limits**
A	Split between Cerotomites / Diabroticites	61.34 Mya	54.92 – 67.76 Mya
B	Split between *Diabrotica* and *Acalymma*	44.64 Mya	37.41 – 53.17 Mya
C	Basal radiation within Cerotomites	31.75 Mya	21.96 – 43.34 Mya
D	Basal radiation within *Diabrotica*	30.76 Mya	25.76 – 36.88 Mya
E	Basal radiation within *Acalymma*	27.29 Mya	21.02 – 33.82 Mya
F	Basal radiation within vigifera group	17.01 Mya	13.41 – 21.10 Mya
G	most recent speciation event	0.48 Mya	0.01 – 1.45 Mya

* as presented in Figure 2.

The mean value for divergence times indicated that the split between Cerotomites and the other Diabroticina subtribes occurred at *ca*. 60 Mya (95% confidence limits 55 - 68 Mya; Figure 2, node A). The split of the common ancestor of *Acalymma* and *Diabrotica* *sensu lato* was dated at *ca*. 45 Mya (95% confidence limits 37–53 Mya; Figure 2, node B). The important radiation events within the genera *Cerotoma*, *Diabrotica*, and *Acalymma*, began almost simultaneously; our estimates place these events at *ca*. 32, 31, and 27 Mya respectively (Figure 2, nodes C, D, and E). Another meaningful evolutionary episode in the history of the genus *Diabrotica* took place around 17 Mya. During that time, a reduction in the diet breadth of Diabroticina took place, resulting in a secondary specialization on host plants, switching from the polyphagous fucata to the oligophagous virgifera group (node F). Several speciation events were dated during the Pleistocene; being the most recent, the divergence between *Diabrotica nummularis* and *Diabrotica biannularis* that occurred *ca*. 500 000 years in the past (node G).

Based on the coalescent analysis we deduced that the main radiation within Diabroticina beetles occurred between middle Oligocene and middle Miocene (Figure 2). *Cerotoma* shows the slowest radiation rate with an average time between nodes of 13 My (Table 4). *Acalymma* has an intermediate radiation rate. This genus underwent on average one evolutionary splitting event almost every 7 My. Finally, *Diabrotica* *sensu lato* is the most specious clade; consequently, this group presents the highest radiation rate. The average time between internodes is 5 My. If we compare the number of lineages through time *Cerotoma* and *Diabrotica* *sensu lato* show a relatively constant increment in diversity. *Acalymma* instead, displays a fast increment in diversity between 27 and 19 My in the past, followed by a 10 My stasis period, and then new radiation events during the last 9 My.

**Table 4.** Comparativerate and timing of speciation among Diabroticinas.<br/>

**Lineage**	**average time**	**S ^a^**	**D ^a^**	**γ ^b^**
*Cerotoma*	13.29 My	0.034	0.057	-0.273
*Acalymma*	7.62 My	0.046	0.111	-1.252
*Diabrotica* *sensu lato*	5.02 My	0.068	0.154	-1.510
Clade I	4.63 My	0.071	0.152	-0.992
Clade II	5.33 My	0.062	0.187	-0.818

**a** Good-Avila et al. 2006

**b** Pybus and Harvey 2000

## Discussion

Diversity patterns change across geography, geological time, and phylogenetic level. Key characters (e.g. flight ability, host specialization, pharmacophagy, etc.) are the central concept of the adaptationist approach that explains how lineages can radiate through those different levels. Species or lineages move into “unoccupied” or new adaptive zones, and thanks to those key characters the lineage goes through a process in which the rate of speciation depends on characteristics of that zone. If we analyze such an adaptationist approach to explain biodiversity based on its theoretical principles we might conclude the following: a) lineages radiate and adapt to different life strategies; b) those strategies are what we call “adaptive zones”; c) the main evolutionary force that mediates speciation (and extinction) rates is Natural Selection; d) this evolutionary force interacts with the fore mentioned key characters, resulting in further lineage adaptation and differentiation. Key characters are usually considered as intrinsic features of the lineage. Nonetheless, speciation rate is regulated by both intrinsic and extrinsic factors. Next we discuss a series of “key features” that may be responsible for modelling the biodiversity patterns observed in Diabroticina beetles.

### Evolutionary radiation: Pharmacophagy

Most wild species of Cucurbitaceae contain bitter, toxic secondary compounds known as cucurbitacins. These tetracyclic triterpenoids are synthesized from mevalonic acid. Cucurbitacins are the bitterest natural molecules known and protect the plants against many herbivores (Torkey et al. 2009). Diabroticina beetles, however, have overcome the deterrent effects of cucurbitacins. Moreover, *Diabrotica* spp. are the most sensitive insects to these phagodeterrents known. With the exception of *Amphelasma cavum*, all Diabroticina species studied to datefeed compulsively as soon as they get in contact with cucurbit tissue. (Eben et al. 1997, Eben and Espinosa de los Monteros 2008). Once ingested, cucurbitacins are fixed in the beetle’s tissues, and may protect the insect against predators and pathogens (Ferguson and Metcalf 1985, Nishida and Fukami 1990, Tallamy et al. 1998, 2000, Gámez-Virués and Eben 2005b). Pharmacophagous lineages, therefore, should have a higher survival and reproduction rate.

To gain access to the compounds, adapted beetles have developed curious behaviours. Morchete (*Cucurbita okeechobeensis martinezii*) leaves frequently show a semicircular cut along their edges. Field observations have demonstrated that a coccinellid beetle (*Epilachna tridecimlineata*) is responsible for this damage. This vein cutting behaviour impedes the coagulation of sticky phloem sap around the insect’s mouthparts. In continuation, the insect starts to feed on the tissues inside the circle. Once the trench is finished Diabroticina beetles begin to feed alongside the coccinellid from the tissue inside the semicircle (Eben and Gámez-Virués 2007). Cucurbitacins are such sequestered in the hemolymph, and fixed in the insect tissue including exoskeleton, and gonads (Ferguson and Metcalf 1985). Thus pharmacophagy not only gives protection to the adults, the bitter substances are also transferred to the eggs repelling potential predators (Brust and Barbercheck 1992). The “chemical defense” obtained by pharmacophagy, however, is questionable. Brust and Barbercheck (1992) observed no differences in the predation rate on eggs laid by females of *Diabrotica undecimpunctata howardi* fed on bitter and non-bitter cucurbits. The bitter eggs, nonetheless, hatched first. This might give the larvae a head start for finding food sources. Gámez-Virués and Eben (2005a), based on laboratory experiments, report that *Repipta flavicans* Stål (Hemiptera: Reduviidae) preyed on adults of *Acalymma blomorum* regardless of the beetles’ diet. Surprisingly, the cucurbitacins contained in the beetles’ tissue were sequestered by the assassin bug; furthermore, these “bitter” bugs lived longer than the bugs fed on non-bitter insects.

Although the hypothesis of sequestering cucurbitacins for the insect’s protection is very appealing, there is data suggesting that Diabroticinas do not receive any fitness benefit from this behavior. On the contrary, some experiments have shown that the metabolic costs are high. For instance, the larvae fed on cucurbitacin containing diet have a lower growth rate than those fed on a cucurbitacin free diet (Ferguson et al. 1985, Hirsh and Barbercheck 1996, Eben and Barbercheck 1996). Detoxification costs, apparently, are correlated with feeding habits and host range. The oligophagous species *Diabrotica virgifera virgifera* had higher costs, than polyphagous taxa (e.g. *Diabrotica balteata*, *Diabrotica undecimpunctata*), but lower than monophagous species like *Acalymma vittatum* (Ferguson et al. 1985).

So far, the available evidence for an advantage of pharmacophagy is inconsistent. More observations and experiments are essential to shed light on the role of pharmacophagy in the evolutionary fate of these beetles.

### Evolutionary radiation: Beetle/host-plant "coevolution"

A lineage that occupies heterogeneous environments (e.g. geographical, ecological, climate, host species) might speciate more rapidly than others that inhabit homogeneous environments. When such is the case, the difference between closely related lineages showing disparate diversities is not due to the expression of key adaptation. It is just the consequence of exploiting environments of different complexity (Cooper and Westneat 2009, Aguilar-Medrano et al. 2011). Therefore, clades that have the ability for using a more diverse environment should have the highest biological diversity. Many species of insects spend their entire live cycle on a single plant. Host plants, therefore, represent the only environment known by those species. Insect/host-plant interactions play a significant role in the long-term evolution of insect lineages, and have exerted reciprocal influences on one another’s diversification (Farrell and Mitter 1994). Detailed co-evolutionary scenarios can be reached after comparing the cladogenetic patterns between interacting lineages. Currently, there are some robust phylogenies for the plants associated with Diabroticina beetles [e.g. Cucurbitaceae (Schaefer et al. 2009), Angiosperms (Magallón and Castillo 2009)]. Unfortunately, the available information of specific hosts used by Diabroticinas is too vague (e.g. beans, corn, etc), and in many cases nonexistent. Therefore, general evolutionary scenarios for diet spectrum and main host invasion are presented in Figures 1 and 2.

Host plant selection depends on the insect’s perception of the rate between stimulant and deterrent compounds in the plants. In the genus *Chrysolina*, host changes are preceded by exploring other closely related plant species (Termonia et al. 2001). Similar behaviour are expected in other polyphagous Diabroticinas. Based on molecular phylogenies several authors have inferred evolutionary scenarios for host plant use in Diabroticina beetles (Szalanski et al. 2000, Clark et al. 2001, Eben and Espinosa de los Monteros 2004). These beetles show a wide range of host breadth: from monophagous species (*e.g*. *Acalymma* spp., *Diabrotica scutellata*, *Isotes teraspilota*, *Paratriarius curtisii*) to polyphagous species that may use more than 300 host species from over 50 families (e.g. *Diabrotica balteata*, *Diabrotica speciosa*). Our scenario (see Eben and Espinosa de los Monteros 2004 for more details) shows that the basal lineages of Diabroticina feed exclusively on one plant family (i.e. Fabaceae); therefore, the ancestral condition is monophagy (Figure 1). Although some lineages have discarded Fabaceae as hosts, the monophagous state was upheld within the Diabroticina lineage, and the oligophagous condition independently evolved twice. Early in the evolution of these beetles Cucurbitaceae was incorporated within their host range, and has been maintained in most species (Figure 2). The genus *Cerotoma* was characterized by a slow increment in the number of hosts, reaching a polyphagous spectrum in some of the apical lineages. Nonetheless, a secondary regression to oligophagy within this genus wasinferred. At the base of the *Diabrotica* clade a fast acquisition of hosts was observed. Throughout the evolution of Diabroticina the use of Poaceae species has independently occurred at least three times. Secondary regressions to oligophagy, monophagy, and also polyphagy were observed in the genus *Diabrotica*. On the other hand the least diverse genus *Acalymma* retained the ancestral condition of monophagy. This scenario supports the idea that the Diabroticina beetles are one example of behavioural plasticity; furthermore, it contradicts the generalist to specialist trend commonly assumed to be the result from the evolutionary process (Kelley and Farrell 1998, Janz et al. 2001).

Ferguson et al. (1985) demonstrated that polyphagous species (e.g. *Diabrotica* *sensu lato*) sequester less cucurbitacin than monophagous lineages (e.g. *Acalymma* spp). Some studies concluded that the optimization of metabolic pathways for the detoxification of cucurbitacins might be the explanation for the secondary monophagy on cucurbits (Andersen and Metcalf 1987, Metcalf and Lampman 1989). Apparently, leaf-beetles show a high ecological plasticity that enables them to switch hosts depending on their availability (Pasteels and Rowell-Rahier 1991). Consequently, the trophic niche that was available after such physiological adaptations could have favored the expansion and subsequent cladogenesis in the New World Diabroticinas.

### Evolutionary radiation: Competitive exclusion

One more process that may have favored the rapid radiation within Diabroticina is the result of ecological interactions with closely related lineages. Taxa that diversify to a large extent during their evolutionary history may fill available ecological space, pushing less fit individuals towards alternative adaptive zones leading to subsequent ecological diversification within subclades. Several evidences have supported such evolutionary pathways. It has been documented that strong competition occurs among the population members of some genera of lizards. In the absence of other sympatric species such competition, apparently, is responsible for the members of these taxa to experience ecological release and niche shifts (Smith 1981, Sites et al. 1992, Knox et al. 2001).

In Mexico, Diabroticina beetles are rarely found feeding on cucurbit leaves (either wild or cultivated; Eben and Barbercheck 1996, Gámez-Virués and Eben 2005b). These insects, however, are abundant inside the male flowers feeding on pollen and possibly waiting for potential mates. Field observations in central Veracruz frequently found more than 20 male insects sitting inside a single flower (Gámez-Virués and Eben 2005b). Similar observations have been reported for other species of Diabroticina in South America (Cabrera-Walsh 2005). Although, in Mexico cucurbit plants are present all year long, the number of flowers per plants is small (less than five per day). Flowers, thus, represent a limited resource for Diabroticina beetles.

An untested hypothesis states that males may be searching for food sources rich in cucurbitacins, because these secondary compounds are transferred to the females within the spermatophors (Eben 2012). Chemical analysis of pollen from *Cucurbita moschata* listed high concentrations of beta-alanine, asparagine, and alanine; however, the presence of secondary compounds like cucurbitacins has not been registered (Mullin et al. 1994). These authors also reported that the amino acids present in the pollen triggered phagostimulant pathways via chemoreceptor cells. When pollen from different plants was offered to adults of several species of *Acalymma* and *Diabrotica*, most insects showed a significant preference for the pollen of Cucurbitaceae (Eben and Van Loon, unpublished data). An interesting result was that species of Diabroticina that have been reported as cucurbit specialists fed on the same amounts of pollen from other plant species like corn. Poaceae has a pollen composition based mainly on proline, alanine, and GABA (Mullin et al. 1994). The preference for certain flowers, therefore, may be mediated by the occurrence of one, or the combination of several amino acids, instead of secondary compounds. It is possible that other factors like scents, form, and color of the flowers are determinant for selecting a specific plant host (Andersen and Metcalf 1987). It is probable, therefore, that those “feeding leks” promote competition pressure and different fitness among the individuals.

Differential selection (natural or sexual) intensities are the causal agent for variable rates of evolution and speciation (Cracraft 1992). However, there is considerable debate about the importance of biotic interactions such as competition in structuring the distribution and abundances of species populations and therefore communities (Mayr 1997). This is a process that has spatial and temporal scales at the level of local populations and their speciation and extinction rates, and thus in structuring phylogenetic diversity. Without doubt, the need for specific studies centering on the effect of intra-specific competition on morphological, physiological, ecological, or behavioral shifts in insects must be considered a priority.

### Evolutionary radiation: Geomorphological complexity

One of the primary determinants of speciation rate is extrinsic, in that it largely interlocks processes external to the lineages that are differentiating. Spatial and long-term temporal variation in geological complexity influences the rate at which populations become isolated and therefore differentiated. Since the evolutionary synthesis, geographic isolation has been regarded as a main factor in promoting taxonomic differentiation within most terrestrial lineages (Mayr 1997).

Mesoamerica, the putative center of origin for Diabroticina beetles, is one of the most complex biogeographical areas in the world (Ferrari et al. 1999, 2000, Morrone 2002). This complexity reflects the confluence of Neotropical and Nearctic ecosystems and a long history of geological activity, stretching from the Late Oligocene to the present (Guzman-Speziale et al. 2005). Throughout this period, movements of the Cocos, North American, Pacific and Caribbean Plates created barriers and land-bridges that have fragmented and merged the distribution, or allow long distance dispersal, of terrestrial populations (Zeh et al. 2003, Ornelas et al. 2013). During the Miocene intensive tectonic processes took place that were responsible for modifying the topographic landscape of Mesoamerica. The Trans Mexican Volcanic Belt and Sierra Madre del Sur were formed at this time (Ferrari 2004). The Pliocene, also, was marked by a number of significant tectonic events. One such event was the joining of the plates of North and South America. This had a significant impact on flora and fauna (Behrensmeyer et al. 1992). Pleistocene climate fluctuations had a deep effect on Middle American populations (Hewitt 2000). The existence of several refuges has been postulated in Mexico (Toledo 1982, Ceballos et al. 2010). Neotropical montane forests experienced extremely complex glacial-interglacial dynamics. The available data describe different scenarios concerning the effect of climatic fluctuations on the genetic structure and population history of species distributed in these habitats (see Ramírez-Barahona and Eguiarte 2013, and references within). Ornelas et al. (2013) documented temporal and spatial genetic divergence of 15 species (including seed plants, birds and rodents), and related them to the evolutionary history of the naturally fragmented cloud forests in Mesoamerica. Their results showed shared phylogeographic breaks that correspond to the Isthmus of Tehuantepec, Los Tuxtlas, and the Chiapas Central Depression. However, the identified barriers are apparently lineage-specific revealing a complexity that seems to be the result of differences among taxa in ecological niche requirements and dispersal capabilities. It is likely that within these habitats there existed multiple successive opportunities for populations to diverge in isolation.

The identification of biogeographic breaks needs to be considered in a temporal framework, which allows comprehension of some of the present day diversity patterns for Diabroticina beetles. Temporal consideration in biogeographic analyses has been neglected in historical biogeography (Avise 2000). However, new methods involving mt DNA analysis could lead to an improvement in the identification of historical scenarios.

### Tempo of evolution

The use of divergence times has been severely criticized due to the presence of different rates of evolution in different taxonomic groups or even in individual genes (Nabholz et al. 2009). This study shows that it is possible to identify temporal congruence, in spite of the different evolutionary rates and divergence sequence within the taxa. Although many of the individual lineages show a sympatric distribution in the present, the individual divergences represent different evolutionary histories. The heterogeneity in those lineages reflects different responses to the same climatic, geological and ecological events that have modelled the actual configuration of their genetic structure, distributions, and biodiversity (Avise 2000). Unfortunately, for most cases, the description of genetic divergence and biodiversity patterns has been established without a temporal framework (Liebherr 1994, Vandergast et al. 2008). Consequently, the difficulties of setting an adequate temporal frame are limited by a gap in the understanding of the evolutionary history of the groups. The use of fossil data, which provides a better estimate of minimum divergence times (Oaks 2011), is confounded in several taxa given the paucity or absence of a fossil record. Nevertheless, a rough estimation based on each taxon’s evolutionary pattern could be useful in the establishment of a temporal framework in the diversity inference of biota (Daza et al. 2010).

While the origin of Diabroticinas could be set sometime during the Cretaceous (Figure 2), our data suggest that the diversification of the Diabroticites probably started *ca*. 62 Mya. This would be just after the Cretaceous-Paleogene boundary, a harsh climatic period of Earth’s history associated with a global biodiversity turnover. The initial radiation process can be attributedto the acquisition of Cucurbitaceae as a new plant host, and consequently to the origin of pharmacophagy. The split and further diversification of the main lineages, however, did not start until the Eocene/Oligocene boundary (ca. 34 Mya). Interestingly, this concurs with the inferred radiation date for other non-related lineages [e.g. Neotropical trogons (31 Mya, Moyle 2005), Neotropical parrots (35 Mya, Schweizer et al. 2011), the Microphyla subsection in the genus *Bursera* (30 Mya, De Nova et al. 2012), *Commiphora* the second most specious genera in Burseraceae (from 30 Mya, Becerra et al. 2012, to 33 Mya, De Nova et al. 2012)]. Metcalf and Lampman (1989) proposed the “inter-cropping theory” in order to explain the high diversity in some lineages of Diabroticites. This theory is based on the prehispanic agricultural tradition of intermixed cropping with corn, beans, cucurbits and chilies. The ancestral Diabroticina invaded those rich spots loaded with potential new hosts. Then, fast switches within the feeding niche led the ancestral species into different adaptive peaks. So, favoring an explosive speciation process that gave origin to the high species diversity observed in this beetle group.

Such Mesoamerican agricultural practices, however, originated *ca*. 10,000 years ago (Smith 1997). The start of the radiation for the three genera surveyed in this study was estimated between 32 and 27 Mya. More precisely, our data shows that the diversification within *Cerotoma* occurred between 18 and 7 Mya, for *Acalymma* between 24 and 5 Mya, whereas for *Diabrotica* between 22 and 0.5 Mya. These radiation periods are incompatible with an explosive radiation mediated by agricultural techniques. There is no doubt that mixed cropping systems and monocultures have privileged the dispersal of rootworm beetles, and in some cases allowed a species to become an important pest. It is not probable, however, that such a level of speciation took place in a short time. Although species sampling is limited in comparisonto the number of extant lineages, the radiation observed in Diabroticina lineages is not recent (i.e. post-Panamanian uplift, 4 Mya). At least five apical nodes indicate recent speciation events in the genus *Diabrotica*. These splits occurred between 1.9 and 0.5 Mya, and might be the result of the Pleistocene climatic oscillation.

As expected, the rate of diversification changed considerably among the mayor lineages of Diabroticina (Table 4). *Cerotoma* was the slowest lineage showing one cladogenetic event every 13.3 My. Whereas, Clade I within the genus *Diabrotica* speciated nearly three times faster (i.e. average splitting time 4.6 My). The rate of diversification inferred in the genus *Cerotoma* is D = 0.057 species per My or approximately a third of the rate observed in Clade II in *Diabrotica* (D = 0.187 species per My). An increment in the diet spectrum going from the oligophagous species of *Cerotoma*, to the polyphagous species of the fucata group encompassed in the different clades of the genus *Diabrotica* might explain such changes in the diversification rate. Other factors, nonetheless, could also be involved within the complex dynamics of species formation. When the phylogenetic pattern is included a similar scenario is observed. Calculations set the highest rate within Clade I (S = 0.071), and the lowest within the *Cerotoma* lineage (S = 0.034). Regardless of the fact that the generation time in these insects is significantly smaller than the generation time of many plants, the S values obtained for Diabroticinas are at least one order of magnitude smaller than those observed in some genera of plants that have undergone rapid events of speciation (e.g. *Agave* *sensu lato*, S = 0.320; Good-Avila et al. 2006). Incomplete sampling of taxa, however, can artificially bias the average time between internodes, as well as the diversification rate indexes (D, and S). A useful approach, thus, is to evaluate the timing of speciation within the lineage. For all the main lineages of Diabroticina the γ statistic took negative values (Table 4). This would suggest that the speciation rates have been slowing down toward the present. None of the γ values are smaller than -1.645; therefore, the null hypothesis of constant birth-death process cannot be rejected at the 5% confidence level (one-tailed test, Pybus and Harvey 2000).

The evolutionary history and biodiversity patterns in the Diabroticina beetles is very complex and has been the result not only of recent climatic oscillation, but the combination of several intrinsic and extrinsic factors. Our data support the conclusion that these insects have gone through a series of dispersion and speciation events that have been the result of events occurred in Mesoamerica since the Eocene until the present. Unfortunately, we did not obtain samples from species belonging to the South American signifera group. Those samples are essential for understanding the biogeographic and diversification history of the genus *Diabrotica*, and for testing the hypothesis that the invasion of South America is a recent event posterior to the Panamanian uplift. Finally, the species sampling must be increased especially for the species rich South American genera in order to corroborate the ideas presented here.
